# Evolutionary engineering of a glycerol-3-phosphate dehydrogenase-negative, acetate-reducing *Saccharomyces cerevisiae* strain enables anaerobic growth at high glucose concentrations

**DOI:** 10.1111/1751-7915.12080

**Published:** 2013-09-04

**Authors:** Víctor Guadalupe-Medina, Benjamin Metz, Bart Oud, Charlotte M van Der Graaf, Robert Mans, Jack T Pronk, Antonius J A van Maris

**Affiliations:** 1 Kluyver Centre for Genomics of Industrial FermentationJulianalaan 67, 2628 BC, The Netherlands; 2 Department of Biotechnology, Delft University of TechnologyJulianalaan 67, 2628 BC, The Netherlands

## Abstract

Glycerol production by *Saccharomyces cerevisiae*, which is required for redox-cofactor balancing in anaerobic cultures, causes yield reduction in industrial bioethanol production. Recently, glycerol formation in anaerobic *S. cerevisiae* cultures was eliminated by expressing *Escherichia coli* (acetylating) acetaldehyde dehydrogenase (encoded by *mhpF*) and simultaneously deleting the *GPD1* and *GPD2* genes encoding glycerol-3-phosphate dehydrogenase, thus coupling NADH reoxidation to reduction of acetate to ethanol. Gpd^–^ strains are, however, sensitive to high sugar concentrations, which complicates industrial implementation of this metabolic engineering concept. In this study, laboratory evolution was used to improve osmotolerance of a Gpd^–^ *mhpF*-expressing *S. cerevisiae* strain. Serial batch cultivation at increasing osmotic pressure enabled isolation of an evolved strain that grew anaerobically at 1 M glucose, at a specific growth rate of 0.12 h^−1^. The evolved strain produced glycerol at low concentrations (0.64 ± 0.33 g l^−1^). However, these glycerol concentrations were below 10% of those observed with a Gpd^+^ reference strain. Consequently, the ethanol yield on sugar increased from 79% of the theoretical maximum in the reference strain to 92% for the evolved strains. Genetic analysis indicated that osmotolerance under aerobic conditions required a single dominant chromosomal mutation, and one further mutation in the plasmid-borne *mhpF* gene for anaerobic growth.

## Introduction

Bioethanol production with *Saccharomyces cerevisiae* is the single largest fermentation process in industrial biotechnology with an annual global product volume of *ca.* 8.6 × 10^10^ l (Renewable Fuels Association, 2012). This puts *S. cerevisiae* at the centre of a global research effort to improve its productivity, robustness under process conditions, substrate range and product yield (van Maris *et al*., 2006). Anaerobic fermentation of sugars to ethanol and CO_2_ is a redox-neutral process. However, in cultures of *S. cerevisiae*, an ‘excess’ of NADH is generated from biosynthetic reactions such as oxidative decarboxylations in amino acid and lipid synthesis (van Dijken and Scheffers, 1986; Bakker *et al*., 2001). In anaerobic yeast cultures, this ‘excess’ NADH is reoxidized through glycerol formation via NADH-dependent reduction of dihydroxyacetone phosphate to glycerol-3-phosphate (G3P), which is subsequently dephosphorylated to glycerol. Glycerol production has been estimated to account for a loss of 4% of the consumed sugar in industrial ethanol production (Nissen *et al*., 2000). Under tightly controlled laboratory growth conditions, where biomass yields are typically higher than in industrial yeast fermentation processes, this percentage can be as high as 10% (Verduyn *et al*., 1990; Nissen *et al*., 2000; Guadalupe Medina *et al*., 2010). Elimination of glycerol formation via metabolic engineering strategies has therefore attracted significant interest (Nissen *et al*., 2000; Nevoigt, 2008; Guadalupe Medina *et al*., 2010; Pagliardini *et al*., 2010; Jain *et al*., 2011).

In *S. cerevisiae*, deletion of the *GPD1* and *GPD2* genes encoding NAD^+^-dependent G3P dehydrogenase (EC 1.1.1.8) eliminates glycerol formation (Björkqvist *et al*., 1997). However, such a double deletion also completely blocks growth under anaerobic conditions unless an external electron acceptor for NADH reoxidation, such as acetoin or acetaldehyde, is provided (Scheffers, 1966; Ansell *et al*., 1997; Björkqvist *et al*., 1997). We recently proposed a metabolic engineering strategy for eliminating glycerol production in anaerobic *S. cerevisiae* cultures that is based on the use of acetic acid as electron acceptor (Guadalupe Medina *et al*., 2010). Acetic acid is a common inhibitor present in plant biomass hydrolysates (Palmqvist and Hahn-Hägerdal, 2000). This strategy encompasses expression of a NAD^+^-dependent (acetylating) acetaldehyde dehydrogenase (EC 1.2.1.10) *mhpF* gene (EMBL: CAA70751) from *Escherichia coli* in a *gpd1Δ gpd2Δ* (Gpd^–^) *S. cerevisiae* strain. After activation of acetate by *S. cerevisiae* acetyl-coenzyme A synthetase (van den Berg *et al*., 1996), the resulting acetyl-coenzyme A can be reduced to ethanol by the combined activity of the NAD^+^-dependent (acetylating) acetaldehyde dehydrogenase and yeast alcohol dehydrogenases. Anaerobic growth of the resulting engineered yeast strain on glucose was coupled to acetate reduction (Guadalupe Medina *et al*., 2010). Although the growth rate was reduced, glycerol production was eliminated, and the ethanol yield increased by 13% relative to that of a *GPD1 GPD2* (Gpd^+^) reference strain (Guadalupe Medina *et al*., 2010).

Glycerol formation is not only crucial for redox balancing in anaerobic cultures of wild-type *S. cerevisiae* but, as its main compatible solute, is also required for osmotolerance. As a consequence, Gpd^–^ *S. cerevisiae* strains are sensitive to high osmotic pressures (Ansell *et al*., 1997). Industrial ethanol production use high sugar concentrations at the start of fermentation processes, which makes osmotolerance of the yeast strains an essential attribute (Albertyn *et al*., 1994; Blomberg and Adler, 1989; Nevoigt and Stahl, 1997). The response of *S. cerevisiae* to high osmolarity is regulated by the high-osmolarity glycerol pathway and involves not only intracellular glycerol accumulation but also regulation of other stress-related genes (Hohmann, 2002). The osmosensitivity of Gpd^–^ strains of *S. cerevisiae* (Ansell *et al*., 1997) can be partly alleviated by introduction of sorbitol-6-P-dehydrogenase and mannitol-1-P-dehydrogenase encoding genes (Shen *et al*., 1999).In such engineered strains, mannitol or sorbitol act as alternative compatible solutes, although growth rates are lower than in wild-type strains (Shen *et al*., 1999). An alternative strategy that does not completely eliminate glycerol formation is the replacement of the *GPD1* or *GPD2* promoters by lower-strength constitutive promoters (Hubmann *et al*., 2011).

Evolutionary engineering is a powerful approach to select for strain variants/mutants with (improved) industrially relevant traits. In evolutionary engineering, regimes for prolonged cultivation are designed such that a selective advantage is conferred to spontaneous mutants that express the trait of interest (Sauer, 2001; Oud *et al*., 2012). Evolutionary engineering not only generates strains with industrially relevant phenotypes, but subsequent analysis of molecular mechanisms responsible for their improved performance also enables reverse engineering of these traits into non-evolved strains (Sauer, 2001; Oud *et al*., 2012).

The goal of the present study was to investigate whether evolutionary engineering enables the isolation of osmotolerant mutants of Gpd^–^ *S. cerevisiae* expressing an *E. coli* (acetylating) acetaldehyde dehydrogenase. The ability of these strains to grow anaerobically with acetic acid as electron acceptor makes it possible to specifically focus on anaerobic evolutionary engineering experiments for improvement of osmotolerance*.* To this end, sequential batch cultivation of the engineered strains was performed under anaerobic conditions and at high sugar concentrations that are relevant for industrial cultivation (Laluce, 1991; Jones *et al*., 1994; Bai *et al*., 2008). After prolonged cultivation under selective conditions, which involved glucose concentrations of up to 1 M, single-colony-derived isolates were characterized in anaerobic bioreactors.

## Results

### Evolutionary engineering for improved osmotolerance

**Fig. 1:**
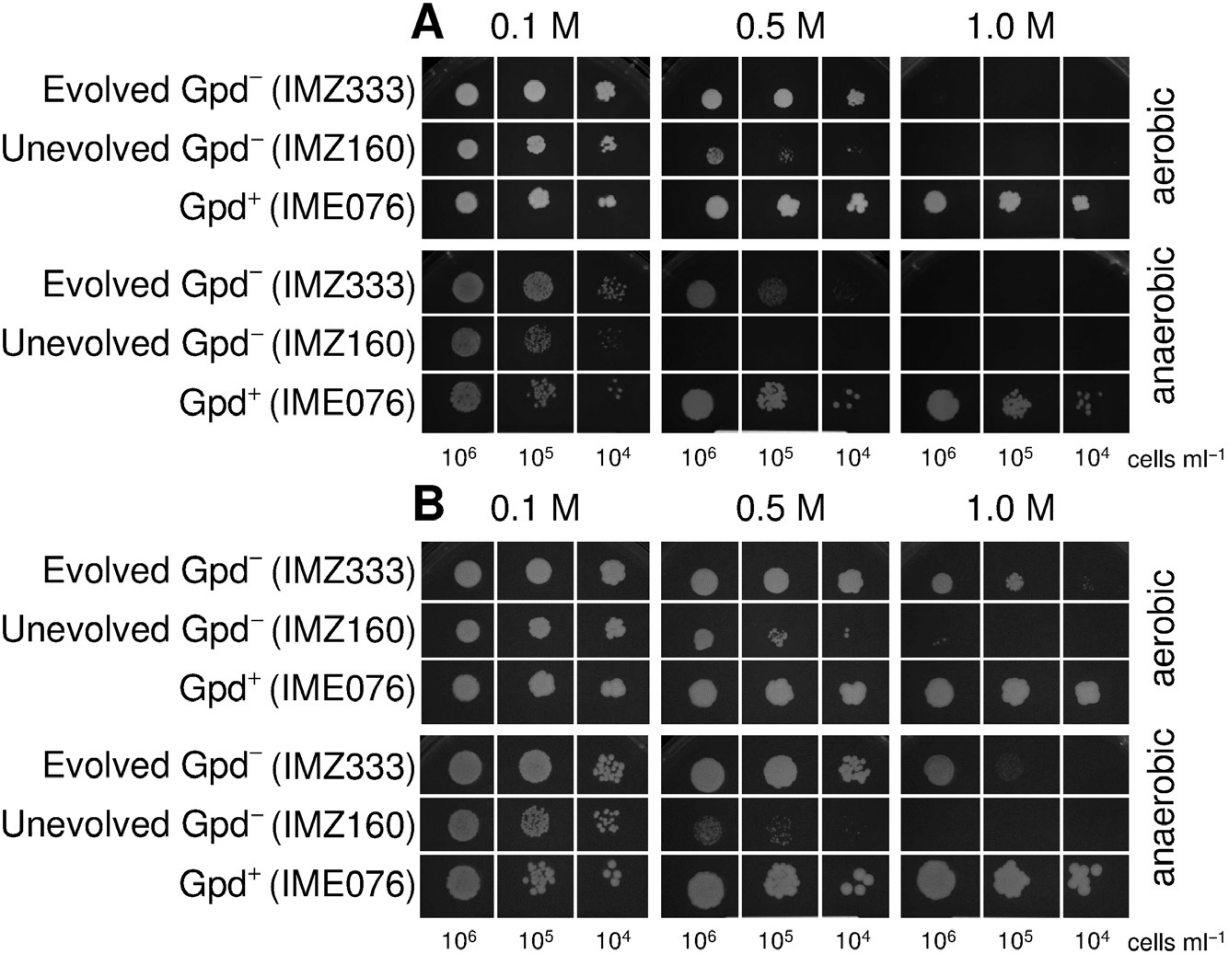
Osmotolerance of evolved strain IMZ333 (evolved Gpd^–^), ancestral strain IMZ160 (unevolved Gpd^–^) and the reference strain IME076 (Gpd^+^). Spot assay experiments were performed on synthetic medium agar plates with 0.1–1.0 M glucose under aerobic and anaerobic conditions. Pictures were taken after 3 days (A) and 7 days (B) of incubation at 30°C.

The ability of *S. cerevisiae* IMZ160 (*gpd1Δ gpd2Δ mhpF*) to grow at industrially relevant osmotic pressures was assessed with spot assays on synthetic medium plates containing 0.1, 0.5 and 1.0 M glucose. In line with previous research on Gpd^–^ strains (Ansell *et al*., 1997), growth of strain IMZ160 was severely inhibited at 0.5 M glucose, both under aerobic and anaerobic conditions, and completely abolished at 1.0 M glucose. Growth of the Gpd^+^ reference strain *S. cerevisiae* IME076 was not inhibited at these glucose concentrations (Fig. 1).

Evolutionary engineering for improved osmotolerance was initiated in shake flask cultures with 20 g l^−1^ glucose as the carbon source supplemented with sorbitol to increase osmolarity. At an initial concentration of 1.0 M sorbitol, the Gpd^–^ strain IMZ160 showed a specific growth rate of 0.06 ± 0.00 h^−1^, as compared with 0.37 ± 0.00 h^−1^ for the Gpd^+^ reference strain IME076. After 4 serial transfers at 1.0 M sorbitol, 8 transfers at 1.5 M sorbitol and 16 transfers at 2.0 M sorbitol, the cultures showed a maximum specific growth rate of 0.18 ± 0.01 h^−1^ at 1.0 M sorbitol and 0.15 ± 0.01 h^−1^ at 2.0 M sorbitol. To achieve anaerobic growth at 1.0 M glucose, a shake flask culture adapted for growth in 2.0 M sorbitol was used as inoculum for an anaerobic bioreactor batch culture at low osmotic pressure in synthetic media with 20 g l^−1^ glucose and 2 g l^−1^ acetate. After 10 days, an anaerobic specific growth rate of 0.12 h^−1^ ± 0.00 h^−1^ was observed. Subsequently, anaerobic sequential batch cultivation was performed on synthetic media supplemented with 2 g l^−1^ acetic acid and with 1.0 M glucose as source of carbon and to increase osmolarity. Starting with an initial anaerobic specific growth rate of 0.05 h^−1^, the sequential batch culture showed a continuously increasing specific growth rate until, after 187 sequential batch cultures, a specific growth rate of 0.13 ± 0.01 h^−1^ was measured as the average of the last 10 sequential batches. Glycerol, which was not detected during the initial cycles, was detected in culture supernatants later in the evolution experiments, albeit at much lower levels than in cultures of the Gpd^+^ reference strain grown under identical conditions (data not shown).

After 187 sequential batch cultures, individual single colony isolates were obtained, whose growth rates were analysed in anaerobic batch cultures on synthetic glucose supplemented with 1.0 M glucose and 2 g l^−1^ acetate. A single-colony isolate that exhibited the highest maximum specific growth rate of 0.12 ± 0.00 h^−1^ and the lowest final extracellular glycerol concentration of 0.64 ± 0.33 g l^−1^ was named IMZ333 (evolved *gpd1Δ gpd2Δ mhpF*). Spot assay experiments under aerobic and anaerobic conditions at 0.1, 0.5 and 1.0 M glucose confirmed that, in contrast to the ancestral Gpd^–^ strain *S. cerevisiae* IMZ160, the evolved strain IMZ333 was able to grow at a concentration of 1 M glucose, albeit slower than the Gpd^+^ reference strain IME076 (Fig. 1).

### Growth and product formation in anaerobic batch cultures at high glucose concentrations

To quantitatively characterize *S. cerevisiae* IMZ333 (evolved *gpd1Δ gpd2Δ mhpF*), this strain was grown in anaerobic bioreactors on synthetic medium supplemented with 2 g l^−1^ acetic acid. At a glucose concentration of 20 g l^−1^, the specific growth rate of strain IMZ333 was 0.21 ± 0.01 h^−1^, which is significantly higher than that of the ancestral Gpd^–^ strain IMZ160 (0.13 ± 0.01 h^−1^), but still lower than the Gpd*^+^* reference strain IME076 (0.32 ± 0.01 h^−1^). Under these conditions, no glycerol formation was observed for either the Gpd^–^ parental (IMZ160) or evolved strain (IMZ333), whereas the Gpd^+^ reference strain produced up to 1.75 ± 0.20 g l^−1^ glycerol.

**Fig. 2:**
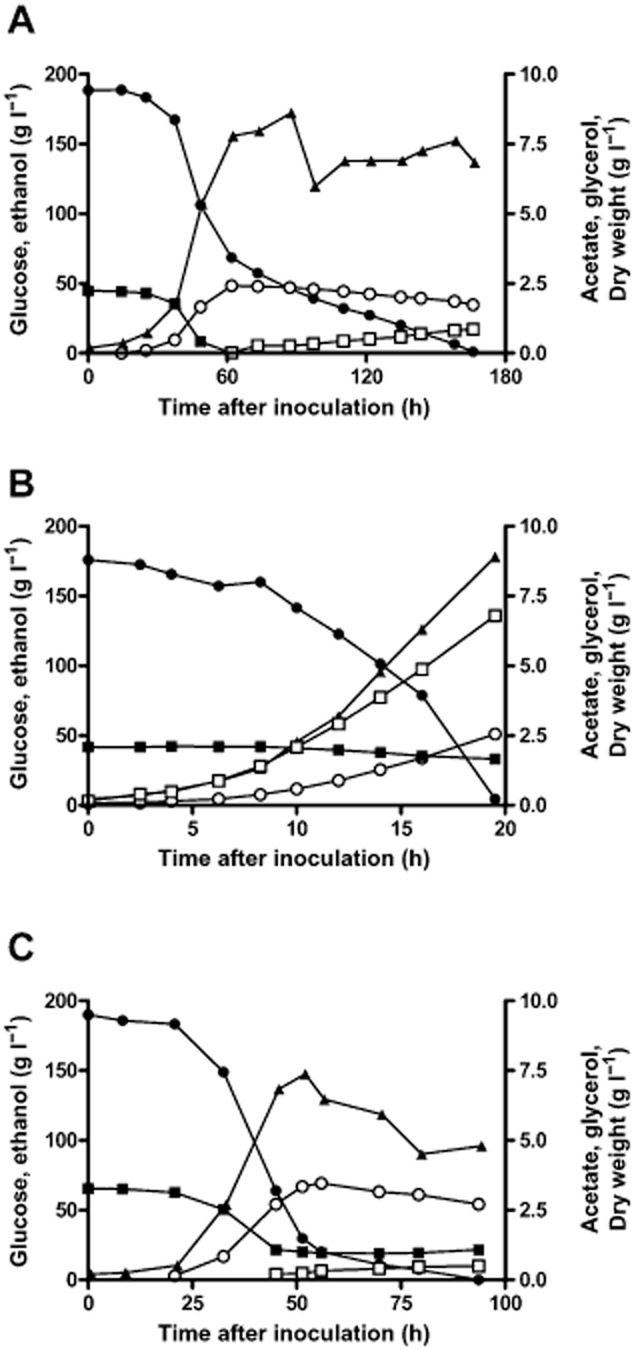
Anaerobic batch cultivation of the evolved osmotolerant strain *S. cerevisiae* IMZ333 (evolved Gpd^–^, A and C) and the reference strain IME076 (Gpd^+^, B) on synthetic medium with 1 M glucose. Both strains were grown at pH 5.0 and at 30°C.A. IMZ333, 2 g l^−1^ acetic acid.B. IME076, 2 g l^−1^ acetic acid.C. IMZ333, 3 g l^−1^ acetic acid. Symbols: ▲, Dry weight; •, glucose; ○, ethanol (not corrected for evaporation); ▪, acetate; □, glycerol. Each graph represents values for one of two independent replicates, which differ less than 5% in growth kinetics.

At an initial glucose concentration of 1.0 M glucose, the evolved strain IMZ333 grew with a specific growth rate of 0.12 ± 0.01 h^−1^ and consumed all sugar in approximately 1 week (Fig. 2A). Under identical conditions, its ancestral strain IMZ160 did not grow during a 10 day incubation, while the Gpd^+^ reference strain grew at 0.24 ± 0.01 h^−1^ and consumed all sugar within 1 day (Fig. 2B).

Growth of the evolved Gpd^–^ strain IMZ333 was clearly coupled to the use of acetic acid as electron acceptor to reoxidize the excess NADH generated during growth (Fig. 2A). During the growth phase, no glycerol was formed by the Gpd^–^ strain. Upon depletion of acetic acid, growth stopped and glucose consumption slowed down. Increasing the acetic acid concentration resulted in a continuation of glucose consumption and in drastically shortened fermentation times (Fig. 2C). When all glucose was consumed, low amounts of glycerol, up to 0.64 ± 0.33 g l^−1^ at 182 h, appeared in the supernatant of cultures of the evolved Gpd^–^ strain IMZ333 (Fig. 2A). However, the glycerol concentration in these cultures remained at least 10-fold lower than those observed in cultures of the Gpd^+^ reference strain (7.4 ± 0.37 g l^−1^ at 19.5 h) (Fig. 2B). The concentration of glycerol measured at the end of the batch cultures supplemented with 3 g l^−1^ acetate (0.53 ± 0.02 g l^−1^ at 93.6 h) was lower than the glycerol concentration measured when 2 g l^−1^ acetate was used (Fig. 2C). Enzyme activity assays in cell extracts of strain IMZ333 confirmed that G3P dehydrogenase activity remained below the detection level of 0.002 μmol min^−1^ (mg protein)^−1^. The activity of (acetylating) acetaldehyde dehydrogenase in IMZ333 was 0.011 ± 0.005 μmol mg protein^−1^ min^−1^, which is not significantly different from the value previously observed for the non-evolved strain IMZ132 [0.020 ± 0.004 μmol mg protein^−1^ min^−1^ (Guadalupe Medina *et al*., 2010)].

The ultimate goal of eliminating glycerol formation in anaerobic yeast cultures is to increase the ethanol yield on sugar. In the nitrogen-sparged anaerobic bioreactors, a significant amount of ethanol is lost through evaporation. Because ethanol loss via evaporation is time dependent, it will be higher for cultures with a lower specific growth rate (Guadalupe Medina *et al*., 2010). After correction for ethanol evaporation, the apparent ethanol yield on glucose of the evolved strain (IMZ333; 1.77 ± 0.09 mol mol^−1^) was 11% higher than that of the Gpd^+^ reference strain (IME076; 1.59 ± 0.02 mol mol^−1^) in cultures grown on 1 M glucose and 2 g l^−1^ acetic acid. At 3 g l^−1^ acetic acid, the apparent ethanol yield of the evolved strain (IMZ333) was 1.84 ± 0.01 mol mol^−1^, which represents 92% of the theoretical ethanol yield on glucose.

### Genetic analysis of the mutations in IMZ333 through mating and sporulation

**Fig. 3:**
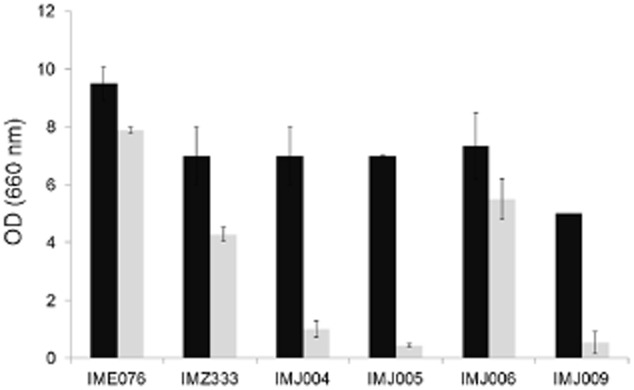
Analysis of the contributions of genomic and/or plasmid based mutations to the evolved osmotolerant phenotype of Gpd^–^*S. cerevisiae*. Aerobic (black bars) and anaerobic (grey-bars) shake flask cultures were both incubated at 30°C and at 200 r.p.m. with an initial glucose concentration of 1 M. The optical density (OD 660 nm) was measured after 48 h for strains IME076 (Gpd^+^ with empty-vector p426_GPD) and IMZ333 (evolved Gpd^–^ with evolved pUDE043 population) or after 72 h for strains IMJ004 (evolved Gpd^–^ pUDE043), IMJ005 (evolved Gpd^–^ and pUDE043ev1), IMJ006 (evolved Gpd^–^ and pUDE043ev2) and IMJ009 (evolved Gpd^–^ with empty-vector p426_GPD).

Mating and sporulation, followed by analysis of segregants, is a powerful approach to investigate the number and nature of mutation(s) in evolved haploid yeast strains (Swinnen *et al*., 2012). To further investigate the evolved osmotolerant genotype, the evolved strain IMZ333 was mated with its osmosensitive ancestral strain IMK006, after transformation with a selection marker and mating-type switching of the latter. Reliable conclusions from crossing and segregation experiments can only be made when the causal mutation(s) reside on the chromosomes rather than on plasmids and when the evolved strain contains the same plasmids as the ancestral strain, thereby avoiding random segregation of different plasmids in the spores. The fact that the acetylating acetaldehyde dehydrogenase activity in the evolved strain (IMZ333) did not increase during the evolution (see above) makes it unlikely that the copy number of the plasmid changed significantly. To assess whether or not there are different versions of the multicopy plasmid present within IMZ333, a subset of 10 plasmids were isolated from IMZ333 and characterized by restriction analysis with XmnI. This indicated that there were at least two types of plasmids in this strain: one that had lost the XmnI restriction site in the *TDH3* promoter upstream of the *mhpF* gene and a second that matched the restriction pattern of the original pUDE043 plasmid. These plasmids were named pUDE043ev1 and pUDE043ev2 respectively. Reinserting these two plasmids and the original pUDE043 plasmid into a plasmid-free ancestral strain IMX031 and in the plasmid-cured evolved strain IMS343 indicated that causal mutations for aerobic osmotolerance were chromosomal because only the evolved strain, transformed with either of the three plasmids, was able to grow aerobically on 1 M glucose plates. Further analysis showed that only the evolved strain with the reintroduced pUDE043ev2 was able to grow anaerobically, albeit at a lower specific growth rate of (0.07 ± 0.01 h^−1^), than the original evolved IMZ333 strain (Fig. 3). This observation indicated that anaerobic growth of the evolved strain on 1 M glucose required chromosomal as well as (a) plasmid-borne mutation(s). Sequencing of the *mhpF* gene on this plasmid revealed a point mutation at base pair position 112 of the open reading frame, resulting in an amino acid change (D38N).

To prevent interference of plasmids in the backcross analysis, the backcross was performed with a plasmid-free ancestral (IMK527) and a plasmid-cured evolved strain (IMS343), and the osmotolerance was tested under aerobic conditions only. The resulting diploid IMD011 was able to grow on a 1 M glucose plate, indicating that the causal mutation(s) conferring aerobic osmotolerance was (were) dominant. Sporulation of this diploid strain revealed a 2:2 segregation of growth on 1 M glucose plates in 19 out of 19 tetrads, indicating that aerobic osmotolerance in the evolved strain is caused by a single mutation (assuming that there are no linked mutations). Also the mutation(s) enabling anaerobic osmotolerance was (were) dominant because the diploid IMD012, resulting from a cross between the ancestral strain IMK527 with the plasmid-containing evolved strain IMZ333, was able to grow anaerobically on 1 M glucose. Sporulation of this diploid strain yielded very few viable spores and no complete tetrads on non-selective medium, which precluded an accurate analysis of the number of causal mutations underlying anaerobic osmotolerance.

## Discussion

We recently proposed a metabolic engineering strategy that enables the use of acetic acid as an electron acceptor for reoxidation of the excess NADH generated in biosynthetic reactions by *S. cerevisiae* and thereby obviates the need for glycerol production for redox balancing (Guadalupe Medina *et al*., 2010). This strategy, which enables increased ethanol yields on sugar, is especially attractive for conversion of lignocellulosic feedstocks, in which acetic acid is invariably present and inhibits yeast fermentation performance. However, the deletion of the *GPD1* and *GPD2* genes encoding G3P dehydrogenase proposed by Guadalupe Medina and colleagues (2010) renders *S. cerevisiae* osmosensitive [(Ansell *et al*., 1997; Guadalupe Medina *et al*., 2010); Fig. 1]. The present study provides a proof of principle that evolutionary engineering can be successfully applied to enable growth of acetate-reducing, *gpd1Δ gpd2Δ* strains to levels that are compatible with industrial bioethanol production. Although further increases in rate are definitely required, this represents an important step towards industrial implementation of an acetate-reducing *gpd1Δ gpd2Δ S. cerevisiae* strain with (acetylating) acetaldehyde dehydrogenase.

The evolved osmotolerant strain (IMZ333) was not only able to grow at 1 M glucose, but converted this sugar to ethanol at increased yields relative to a *GPD1 GPD2* reference strain (11 and 15% increases in cultures grown at 2 and 3 g l^−1^ acetic acid respectively). Part of the increased ethanol yield arises from the elimination of glycerol formation (80 mM in the reference strain), which frees up additional glucose that can be converted to ethanol. A further increase of the ethanol yield can be attributed to the slower growth and longer duration of the fermentation, which increases the fraction of sugar that is converted to ethanol by increasing the cellular maintenance energy requirement (Verduyn *et al*., 1990; Boender *et al*., 2009). Finally, additional ethanol is produced during the reduction of acetic acid, which when all acetic acid is consumed leads to maximal increases of 33 and 55 mM for the fermentations containing 2 and 3 g l^−1^ respectively. The complete consumption of acetate in the cultures grown at 2 g l^−1^ acetate (Fig. 2) illustrates that the (acetylating) acetaldehyde dehydrogenase strategy not only increases ethanol yields, but also enables the detoxification of significant amounts of inhibiting acetic acid. This detoxifying effect may be particularly relevant when high initial acetate concentrations are prevented by gradual feeding, for example by simultaneous saccharification and fermentation or by fed-batch feeding of hydrolysates to yeast fermentation processes (Taherzadeh *et al*., 2001; Rudolf *et al*., 2005).

Even though both genes encoding G3P dehydrogenase were deleted and the enzymatic activity of G3P dehydrogenase was confirmed to be below the detection limit, low concentrations of glycerol were observed upon cessation of growth of the evolved strain IMZ333. Glycerolipids are essential for the growth of *S. cerevisiae* and are formed by acylation of G3P (Racenis *et al*., 1992). However, glycerolipids can also be obtained by acylation of dihydroxyacetone phosphate (DHAP) by the same G3P/DHAP acyltransferase, producing acyl-DHAP, which is later reduced to acyl-G3P by 1-acyldihydroxyacetone-phosphate reductase (EC 1.1.1.101), encoded by *AYR1* (Athenstaedt and Daum, 2000). Through this route, Gpd^–^ *S. cerevisiae* strains are able to form glycerolipids and grow. The low concentration of glycerol that was observed in the evolved Gpd^–^ strain at the end of the fermentation might be formed by deacylation of the glycerolipids and subsequently released when growth stopped and/or cells lysed.

Analysis of the evolved strain IMZ333 indicated that few mutations were required to increase osmotolerance in *gpd1Δ gpd2Δ* acetate-reducing *S. cerevisiae* strain. Detailed analysis of the molecular basis of improved osmotolerance in evolved acetate-reducing strains by whole genome resequencing, thereby enabling its reverse engineering into industrial strains, will require additional, independent evolution experiments to facilitate the separation of causal and random mutations (Oud *et al*., 2012). Such experiments should also reveal whether evolutionary engineering always leads to the low-level *GPD1/GPD2*-independent glycerol formation found in strain IMZ333, or that alternative pathways, for example involving trehalose or proline as alternative compatible solutes (Hounsa *et al*., 1998; Takagi, 2008; Mahmud *et al*., 2009), can also contribute to evolution of increased osmotolerance in these strain backgrounds.

Although analysis of the molecular basis of improved tolerance was outside its scope, the present study provides valuable information for experimental design towards this goal. Firstly, our results indicate that mutations which confer osmotolerance under aerobic conditions are not necessarily sufficient to enable growth at high glucose concentrations in anaerobic cultures. In view of the envisaged application of strains in anaerobic bioethanol processes, future experiments should therefore preferably be performed under anaerobic conditions from the start of the evolution. Secondly, our results indicate that mutations on the *mphF* expression plasmid contributed to anaerobic osmotolerance. To facilitate the application of classical genetics and whole genome resequencing, it is therefore preferable to perform future evolution experiments with engineered strains in which the *mhpF* expression cassette has been integrated into the *S. cerevisiae* genome. Moreover, the identified point mutation indicates that mutagenesis of *mhpF* and/or expression of other (acetylating) acetaldehyde dehydrogenase genes may contribute to osmotolerance in anaerobic cultures.

## Experimental procedures

### Strain construction and maintenance

All *S. cerevisiae* strains in this study (Table 1) originate from the CEN.PK family (van Dijken *et al*., 2000; Entian and Kötter, 2007; Nijkamp *et al*., 2012). Stock cultures and precultures were grown as described previously (Guadalupe Medina *et al*., 2010). *S. cerevisiae* IMK006, obtained by removing the *KanMX* marker from the *gpd1Δ gpd2Δ* strain RWB0094 (Guadalupe Medina *et al*., 2010) by expression of Cre recombinase (Güldener *et al*., 1996), was transformed with the *LEU2*-bearing integrative vector pRS405, which was linearized with BstEII (NEB, Ipswich, MA, USA), yielding the leucine-prototrophic strain IMX031. Transformation of strain IMX031 with the *URA3*-bearing *mhpF*-expression plasmid pUDE43 (Guadalupe Medina *et al*., 2010) yielded the prototrophic, Gpd^–^ *mhpF*-expressing strain IMZ160. Plasmid(s) were isolated from *S. cerevisiae* IMZ333 with the Sigma GenElute plasmid miniprep kit (Sigma-Aldrich Chemie Gmbh, Munich, Germany) according to manufacturer's instructions. Plasmids were transformed into *E. coli* One Shot TOP10 Z-competent cells (Invitrogen, Paisley, UK), and transformants were selected on Luria-Bertani medium plates containing ampicillin (100 mg l^−1^). Restriction analysis of isolated plasmids was performed with XmnI (Fermentas Gmbh, Sankt Leon-Rot, Germany). Plasmid sequencing was performed by BaseClear (Leiden, the Netherlands). *URA3*-bearing plasmids were cured from strain IMZ333 by growth in yeast peptone (YP; 10 g l^−1^ yeast extract and 20 g l^−1^ peptone) medium with 20 g l^−1^ glucose and subsequent selection on YP medium with 5-fluoroorotic acid (5-FOA). A single colony was isolated on synthetic medium containing 20 g l^−1^ glucose, 5-FOA (1 g l^−1^) and uracil and named IMS343. Strain IMJ004 was constructed by transforming strain IMS343 with the original pUDE43 plasmid. Strains IMZ380 and IMZ381, and IMJ005 and IMJ006 were constructed by transforming pUDE043ev1 and pUDE043ev2 into the unevolved parent strain IMX031 and the evolved plasmid-cured strain IMS343 respectively. Strain IMJ009 was constructed by transforming plasmid p426_GPD (*URA3*) into IMS343. Strains bearing plasmids with auxotrophic markers were plated on synthetic media (Verduyn *et al*., 1990; Pronk, 2002) agar plates (1% w/v) using 20 g l^−1^ glucose as carbon source. Confirmation of correct genetic modification and yeast transformations were performed as described earlier (Guadalupe Medina *et al*., 2010).

**Table 1:** *Saccharomyces cerevisiae* strains used in this study

Strain	Relevant genotype/description	Source/reference
IME076	*MAT***a** *ura3 LEU2 GPD1 GPD2* p426_GPD(pTDH3::CYC1t *URA3* 2μ)	Guadalupe Medina *et al*. 2010
RWB0094	*MAT***a** *ura3 leu2 gpd1::loxP-KanMX-loxP gpd2::hphMX4*	BIRD Engineering, Rotterdam, Guadalupe Medina *et al*. 2010
IMK006	*MAT***a** *ura3 leu2 gpd1::loxP gpd2Δ::hphMX4*	This study
IMX031	*MAT***a** *ura3 leu2::LEU2[pRS405] gpd1::loxP gpd2::hphMX4*	This study
IMZ132	*MAT***a** *ura3 leu2 gpd1::loxP gpd2::hphMX4* pUDE43(pTDH3::*mhpF(E. coli)*::CYC1t *URA3* 2μ) YEplac181(*LEU2*)	Guadalupe Medina *et al*. 2010
IMZ160	*MAT***a** *ura3 leu2::LEU2[pRS405] gpd1::loxP gpd2::hphMX4* pUDE43(pTDH3::*mhpF(E. coli)*::CYC1t *URA3* 2μ)	This study
IMZ333	IMZ160 evolved for anaerobic growth at 1 M glucose	This study
IMS343	IMZ333 cured of plasmid	This study
IMZ380	IMX031 with pUDE43ev1(pTDH3:: *mhpF(E. coli)*::CYC1t *URA3* 2μ evolved)	This study
IMZ381	IMX031 with pUDE43ev2(pTDH3*:: mhpF(E. coli)::*CYC1t *URA3* 2μ evolved)	This study
IMJ004	IMS343 with pUDE43(pTDH3*:: mhpF(E. coli)::*CYC1t *URA3* 2μ)	This study
IMJ005	IMS343 with pUDE43ev1(pTDH3*:: mhpF(E. coli)::*CYC1t *URA3* 2μ evolved)	This study
IMJ006	IMS343 with pUDE43ev2 (pTDH3*::mhpF(E. coli)::*CYC1t *URA3* 2μ evolved)	This study
IMJ009	IMS343 with p426_GPD(pTDH3::CYC1t *URA3* 2μ)	This study
IMK527	*MAT*α *ura3::loxP-kanMX-loxP leu2 gpd1::loxP gpd2::hphMX4*	This study
IMD011	Diploid strain resulting from IMS343×IMK527	This study
IMD012	Diploid strain resulting from IMZ333×IMK527	This study

### Shake flask cultivation

Shake flask cultivation was performed as described previously (Guadalupe Medina *et al*., 2010) using synthetic media (Verduyn *et al*., 1990). All shake flaks cultures were grown at 30°C in an Innova incubator shaker (New Brunswick, NJ, USA) at 200 r.p.m. For serial shake flask cultivation, synthetic media with urea as the nitrogen source were used (Verduyn *et al*., 1990). Glucose and sorbitol were autoclaved separately at 110°C and sterile, 10-fold concentrated synthetic medium was added afterwards. Three parallel evolution experiments were performed by serial transfer in aerobic shake flasks. One millilitre from a shake flask preculture of IMZ160 was used to inoculate a first shake flask containing 1 M sorbitol. Serial transfer was performed with 1 ml inocula from shake flasks that had become turbid and reached cell density (OD_600_) higher than 1. At the end of the evolution experiment, a sample of the evolving population was stored at −80°C.

Anaerobic shake flask cultures for inoculum or characterization were incubated in a BactronX anaerobic chamber (Shell Lab, Cornelius, OR, USA) at 30°C and 200 r.p.m. (Heidolph Unimax 2010 shaker; Heidolph, Schwabach, Germany).

### Sequential batch reactors (SBR) and physiological characterization in batch bioreactors

Anaerobic bioreactor batch cultures, off-gas and metabolite analysis, enzymatic glycerol determination, optical density readings, determination of dry weight and enzymatic activity measurements for NAD^+^-dependent (acetylating) acetaldehyde dehydrogenase and G3P dehydrogenase were performed as described previously (Guadalupe Medina *et al*., 2010). All fermentations were carried out at least in duplicate. To correct for ethanol evaporation during cultivation in nitrogen-sparged bioreactors, evaporation kinetics were analysed as described previously (Guadalupe Medina *et al*., 2010).

Sequencing batch reactors were operated as described previously (Wisselink *et al*., 2009). The medium vessels were prepared by autoclaving 18 l of demineralized water containing glucose (1.11 M), and subsequently adding 2 l of 10-fold concentrated synthetic media containing acetic acid (20 g l^−1^), antifoam [0.2 g l^−1^, Emulsion C (Sigma-Aldrich, Zwijndrecht, the Netherlands)], ergosterol (0.1 g l^−1^) and Tween 80 (4.2 g l^−1^). The pH of the 10-fold concentrated synthetic medium containing acetic acid was adjusted to pH 4.8 with potassium hydroxide before autoclaving. A control routine was programmed in MFCS/win 3.0 (Sartorius AG, Göttingen, Germany) to initiate the switch to a new batch cycle. Fermenters were automatically emptied, leaving *ca.* 1.5 ml of remaining culture volume, and refilled when the CO_2_% in the off gas reached 1.2%. When growth accelerated after the first three cycles, this threshold was gradually increased to 3.2% CO_2_.

Single colony isolates were obtained by streaking a sample taken from the SBR on synthetic media agar plates (1% w/v) containing 1 M glucose as carbon source, 2 g l^−1^ acetic acid and anaerobic growth factors. The plates were placed under anaerobic environment in a BactronX anaerobic chamber (Shell Lab) and kept at 30°C. After two transfers of single isolates to fresh agar plates, one colony was inoculated in 1 M glucose synthetic media for stock and named IMZ333. Before characterization in bioreactors, the evolved strains IMZ333 and IMJ006 were precultured anaerobically in synthetic media shake flasks with 1 M glucose as carbon source.

### Spot assays to test osmotolerance

Growth under high osmotic stress was assessed by spotting 5 μl of serial dilution of 10^6^, 10^5^, 10^4^ cells ml^−1^ of exponentially growing cultures onto 0.1, 0.5 and/or 1 M glucose synthetic media agar plates (1% w/v). Overnight shake flasks cultures grown as described before (Guadalupe Medina *et al*., 2010) were used to measure cell density using a Z-Coulter Counter (Beckman Coulter, Brea, CA, USA) and prepare appropriate dilutions in sterile demineralized water. Agar plates were prepared by autoclaving separately glucose and agar from concentrated synthetic media, and mixed thoroughly before pouring the plates. After spotting, all plates were incubated at 30°C under anaerobic and aerobic conditions for 7 days, and pictures were taken at 3 and 7 days.

### Backcrossing and sporulation

To enable crossing, *MAT***a** strain IMK006 was transformed with the marker gene *KanMX* using primers for the *ura3* locus (URA3-KanMXF TTCTTAACCCAACTGCACAGAACAAAAACCTGCAGGAAACGAAGATAAATCCAGCTGAAGCTTCGTACGC and URA3-KanMXR AGCTCTAATTTGTGAGTTTAGTATACATGCATTTACTTATAATACAGTTTTCTTTAAACACGGCCGCATAG) before the mating was switched by transforming plasmid pHO (Herskowitz and Jensen, 1991; Sugawara and Haber, 2012) into this strain. The resulting diploid strain was sporulated yielding a *MAT*α strain IMK527. Sporulation was performed as described by Bahalul and colleagues (2010). Strains were inoculated in YP medium with 10 g l^−1^ acetate as carbon source. After incubation at 30°C for 24 h, cultures were washed and resuspended in sporulation medium (20 g l^−1^ potassium acetate). After 48 h at 30°C, spore formation was checked microscopically. Prior to dissection, a culture sample (1 ml) was incubated with 2 μl zymolyase (1000 U ml^−1^) in a 200 μl 0.5 M sorbitol solution at 37°C for 10 min. Tetrad dissection on YP media plates with 20 g l^−1^ glucose was performed with a dissection microscope (Singer MSM System 300, Singer Instruments, Somerset, UK). Plates were incubated at 30°C. IMK527 (*MAT*α) was crossed with haploid strains IMS343 (*MAT***a**) and IMZ333 (*MAT***a**) by streaking cultures over each other on selective synthetic medium agar plates containing G418 (100 mg l^−1^) on which only diploids could grow. For auxotrophic diploids, uracil (20 g l^−1^) was added to the medium. The resulting diploids were re-streaked, and single colonies were isolated, yielding strain IMD011 (IMS343×IMK527) and IMD012 (IMZ333×IMK527). Sporulation and tetrad dissection of IMD011 and IMD012 were performed as described above. Dissected spores were replica plated on 1 M glucose synthetic medium agar plates to score for osmotolerant segregants.
